# Involvement of loved ones in the rehabilitation process for severe neurotrauma in children

**DOI:** 10.1192/j.eurpsy.2025.1546

**Published:** 2025-08-26

**Authors:** Y. Afanasyeva, M. Bratkova

**Affiliations:** 1 Moscow City University; 2 Clinical and Research Institute of Emergency Pediatric Surgery and Trauma (CRIEPST), Moscow, Russian Federation

## Abstract

**Introduction:**

Among children with developmental disabilities, there is a group with consequences of neurotrauma that requires comprehensive support at different age stages, with the obligatory inclusion of the family in the rehabilitation process. These children are shown both medical and psychological and pedagogical rehabilitation, the effectiveness of which is determined by the active position of the patient’s relatives.

**Objectives:**

The study the factor of involvement of close patients with neurotrauma in the rehabilitation process: manifestations of psychophysical activity.

**Methods:**

140 children with neurotrauma consequences with neuropsychiatric disorders (2021-2024) and 136 families. Medical and pedagogical method with observation, examination, diagnostic and typological assessment of children’s mental activity; cluster analysis of the results of studying families (parents) as participants in rehabilitation.

**Results:**

Clusters were identified based on the assessment of the activity of parents’ inclusion in the rehabilitation process:

Cluster 1. Active families (40%). The patient’s relatives follow all the instructions of the specialists, are an important and integral part of the rehabilitation team. Children from the families studied show both qualitative and quantitative gains in rehabilitation, predominantly in the socio-communicative and cognitive domains.

Cluster 2. Estranged families (60%). Adults formally relate to rehabilitation activities. In children from these families, the dynamics of recovery is limited to an insignificant increase in psychophysical activity indicators, without transition to a higher level.

**Conclusions:**

The effectiveness of the rehabilitation process for severe neurotrauma in childhood is influenced by the involvement of adults close to the child. The personality traits of the parents, adaptability and resistance to stress, the severity of the child’s illness - these factors turn out to be decisive. Most loved ones need medical and psychological support. Regardless of the severity of neurotrauma in children, parents become more active if they note the dynamics in improving mental health. In case of long-term, severe illness of children accompanied by disability, family members only provide care and supervision.

**Disclosure of Interest:**

None Declared

